# Measures of prefrontal functional near-infrared spectroscopy in visuomotor learning

**DOI:** 10.1007/s00221-021-06039-2

**Published:** 2021-02-02

**Authors:** Angelica M. Tinga, Maria-Alena Clim, Tycho T. de Back, Max M. Louwerse

**Affiliations:** Department of Cognitive Science and Artificial Intelligence, Tilburg University, Dante Building, Room D 330, Warandelaan 2, 5037 AB Tilburg, The Netherlands

**Keywords:** Visuomotor learning, Near-infrared spectroscopy (fNIRS), Prefrontal cortex, Feedback

## Abstract

Functional near-infrared spectroscopy (fNIRS) is a promising technique for non-invasively assessing cortical brain activity during learning. This technique is safe, portable, and, compared to other imaging techniques, relatively robust to head motion, ocular and muscular artifacts and environmental noise. Moreover, the spatial resolution of fNIRS is superior to electroencephalography (EEG), a more commonly applied technique for measuring brain activity non-invasively during learning. Outcomes from fNIRS measures during learning might therefore be both sensitive to learning and to feedback on learning, in a different way than EEG. However, few studies have examined fNIRS outcomes in learning and no study to date additionally examined the effects of feedback. To address this apparent gap in the literature, the current study examined prefrontal cortex activity measured through fNIRS during visuomotor learning and how this measure is affected by task feedback. Activity in the prefrontal cortex decreased over the course of learning while being unaffected by task feedback. The findings demonstrate that fNIRS in the prefrontal cortex is valuable for assessing visuomotor learning and that this measure is robust to task feedback. The current study highlights the potential of fNIRS in assessing learning even under different task feedback conditions.

## Introduction

Learning processes are most commonly examined using behavioral measures (Luu et al. 2009; Webb et al. 1966). In addition to behavioral changes taking place during learning, important changes take place in the brain. Cognitive adaptation takes place during learning with connectivity within brain networks and activity in specific brain areas being altered (Bassett et al. 2011; Nackaerts et al. 2019). The advantage of measurements of brain activity is that these measures provide insight not only into the outcome of learning but also into the learning process itself (Tinga et al. 2019, 2020b).

Brain activity can be measured non-invasively through brain imaging techniques that record cortical activity. Effects of learning on changes in cortical brain activity have been reported most frequently at parietal and frontal sites (Tinga et al. 2019). Regarding effects at frontal sites, the prefrontal cortex (PFC) may be an area of special interest for examining learning, as this brain area is involved in cognitive control over and coordination of thoughts and actions (Ayaz et al. 2011) and mediates most higher cognitive functions including learning new skills, rules and behavior (Leff et al. 2011; Wood and Grafman 2003). Additionally, the PFC has been linked to visuomotor sequence learning (Leff et al. 2011). During visuomotor sequence learning the correct order of movements needs to be acquired and executed while simultaneously optimizing sensorimotor parameters such as the trajectory, timing and velocity of the movement (Penhune and Steele 2012). This type of learning plays an essential role as we acquire motor skills in our daily life (Moisello et al. 2009). Visuomotor sequence learning is often contrasted with motor adaptation learning in which a learned movement needs to be adapted to a changed environment (Penhune and Steele 2012). To measure visuomotor sequence learning in the lab, several experimental tasks have been developed. The most commonly applied paradigm in research is the serial reaction time task (SRT) (Nissen and Bullemer 1987). In this task, participants respond to stimuli successively appearing at different locations by making a spatially corresponding response. Unknown to the participants, the responses that need to be made follow a continuous complex sequence. Generally, performance on the SRT, conventionally measured through a number of correct responses and reaction times, improves over time, both due to implicitly learning the sequence of responses and due to learning the principles of the task. Regarding PFC activity during visuomotor learning, activity generally decreases as the task becomes less cognitively demanding and its execution becomes more automatic (Bassett et al. 2011; Wu et al. 2004).

A promising technique for assessing PFC activity in visuomotor learning is functional near-infrared spectroscopy (fNIRS), as this cortical area is imminently accessible using fNIRS (Leff et al. 2011). fNIRS measures cortical hemodynamic activity through the use of near-infrared light. Specifically, near-infrared light is shone on the scalp, whereupon light absorption is measured and hemodynamic activity is inferred from attenuations in light levels (Leff et al. 2011). This technique is therefore based on the assumption that neural activation and dynamics of blood flow are coupled (León-Carrión and León-Domínguez 2012). fNIRS is safe, non-invasive, portable and, compared to other imaging techniques, relatively robust to head motion, ocular and muscular artifacts and environmental noise (Balardin et al. 2017a, b). These properties make fNIRS a promising and versatile tool for studying brain activation not only in the laboratory but also in more natural settings. Yet, fNIRS is applied relatively infrequently as it is still a rather new measurement technique (Kopton and Kenning 2014). In our meta-analysis (Tinga et al. 2019) on studies examining neurophysiological changes during learning within an 11-year window we found 69 experiments incorporating electroencephalography (EEG) measures of brain activity compared to only four experiments using fNIRS. EEG has a temporal resolution superior to fNIRS, yet the spatial resolution of fNIRS is superior to EEG (Crosson et al. 2010; Zama and Shimada 2015). With fNIRS having its own strengths, it is surprising that the number of studies examining fNIRS is relatively low. Another systematic review on motor processes and fNIRS (Leff et al. 2011) only reported five studies examining visuomotor learning using fNIRS, of which three reported effects in the PFC. While fNIRS holds promise in assessing (visuomotor) learning, it is clear there is a need for more studies examining this technique in learning (Leff et al. 2011; Tinga et al. 2019).

Measures of brain activity appear to be sensitive to task-related aspects such as the presentation of task feedback informing the trainee on performance. A considerable number of studies have examined the effect of task feedback on brain activity measured through EEG, demonstrating differences between for example positive and negative feedback(Arbel et al. 2014; Fairclough and Roberts 2011; Opitz et al. 2011; Venables and Fairclough 2009), immediate feedback or feedback with a short delay (Opitz et al. 2011), and feedback in younger and older adults (Eppinger and Kray 2011; Eppinger et al. 2008). Yet, to the best of our knowledge, studies examining the effects of task feedback during learning on fNIRS outcome measures are non-existent.^1^ Consequently, in addition to the need for studies examining fNIRS in learning, it is important to explore the effects of task feedback during learning on fNIRS outcome measures.

In light of this need for studies, the goal of the current study is to assess PFC activity using fNIRS during visuomotor learning and examine how results are affected by feedback. In a recent study (Tinga et al. 2020a) we experimentally manipulated whether participants were provided with direct feedback on task performance (Feedback) or not (No-Feedback) during a visuomotor sequence learning task. Additionally, half of the participants were presented with a switch in Feedback to No-Feedback or the other way around, while no switch occurred for the other half of the participants. A range of neurophysiological outcomes were measured including skin conductance level, heart rate, heart rate variability, respiration rate, eye tracking metrics and brain activity assessed through EEG. Changes during learning in these outcome measures were sensitive to feedback and especially to whether a switch in feedback occurred, while changes in behavioral outcome measures were not. Regarding EEG outcome measures specifically, only a general effect of Feedback versus No-Feedback on alpha power was found, demonstrating a higher power with No-Feedback, suggesting lower cognitive effort investment without feedback during the task in general. Changes in EEG during task performance, which would be reflective of learning, were, however, not affected by (a change in) feedback. To gain insight into how brain activity as measured through fNIRS is affected by learning and how this is affected by feedback, the current study applies the same task as this previous study to examine (1) changes in brain activity during visuomotor learning in activity in PFC measured using fNIRS, (2) whether these changes are affected by feedback and changes in feedback and (3) whether PFC activity assessed through fNIRS provides insight into behavioral learning.

We expected PFC activity to decrease and to be related to behavioral performance in the course of visuomotor learning, which would be in line with the findings on a range of neurophysiological outcome measures including EEG in a recent study by Tinga et al. (2020b) and in line with the notion that less cognitive effort needs to be exerted over the course of learning with behavioral performance improving over time as explained in Tinga et al. (2020c). Changes in PFC activity as assessed through fNIRS could be stronger when No-Feedback is presented. This would be in line with the findings on neurophysiology and brain activity in general in the meta-analysis reported in Tinga et al. (2019) and experimental work demonstrating EEG to be sensitive to feedback as reported in Arbel et al. (2014), Fairclough and Roberts (2011), Opitz et al. (2011) and Venables and Fairclough (2009). It would also be in line with the notion that performance might be supported through feedback (Faulkner et al. 2011), leading to a stronger decrease in cognitive effort exertion over the course of learning. Alternatively, these changes may be unaffected by feedback, in line with the findings on EEG specifically in Tinga et al. (2020a).

## Methods

### Participants

Forty-two (28 female, 14 male) students at Tilburg University participated in the experiment of the current study. Participants were on average 21.40 (SD = 2.44, range 18–26) years old. Participants were included if they reported no current cardiovascular disease, neurological disorder, and lung disease (following Tinga et al. 2020a, b). None of the participants reported to be colorblind. The study was approved by the Research Ethics Committee of Tilburg School of Humanities and Digital Sciences and was carried out in accordance with the Declaration of Helsinki. Informed consent was obtained from all individual participants included in the study.

### Apparatus and measures

The task was presented on a desktop monitor (BenQ Zowie XL2540, 1920 × 1080 pixels, 240 Hz refresh rate) using Unity 3D (version 2017.4.1). Participants used a joystick (Ultimarc UltraStik 360, mounted on the table 16 cm in front of the subjects’ body midline) to interact with the task. The coordinates of the position of the cursor were recorded at 90 Hz.

A BIOPAC 100 W (BIOPAC Systems Inc, USA) continuous wave wireless fNIRS device was used to measure PFC activity at 4 Hz. The device consisted of two sensor pads with each one LED light source emitting near-infrared light at 730 nm and 850 nm wavelengths, which are absorbed primarily by deoxygenated and oxygenated hemoglobin respectively. The sensor pads also each had two light detectors (silicon photodiodes with integrated trans-impedance preamp), thus resulting in a total of four recording channels. The inside of both sensor pads was aligned with the nasion and the pads were placed right above the eyebrows, which would result in measurements of brain activity from the dorsal and inferior frontal cortical areas (Ayaz et al. 2006). Care was taken to avoid hair interfering with the light detectors and sources. fNIRS data was recorded in the software program Cognitive Optical Brain Imaging (COBI) Studio (fNIR Devices, Potomac, MD, USA).

### Learning task and stimuli

The learning task was comparable to the one employed in Tinga et al. (2020a, b) which is a version of SRT in which responses to targets needed to be made using arm movements. The target stimuli to which participants needed to respond were eight white circles presented on a dark gray background. The circles had a diameter of 108 pixels and were evenly spaced apart at 360 pixels from the center of the screen. Additionally, a white circle with a diameter of 108 pixels was presented in the center of the screen.

The learning task consisted of two parts. In both parts, participants moved the cursor (black small circle with a diameter of 33 pixels) with the joystick from the middle of the screen to one of the targets and back to the middle. The target for the movement was selected by one of the white circles turning light gray. Target selection was always in synchrony with a 160 ms tone (presented via headphones) at an interval of 1 s. The first and the second part began with 16 and 8 practice trials respectively and each part consisted of 4 learning blocks, each with 128 trials of 1 s. Targets for movements were selected in a repeating sequence of 16 elements in which each target was selected twice. Two of such sequences were used, one in each of the two parts, with the order of the two counterbalanced between participants.

In each of the two parts of the task feedback was provided in each trial (Feedback), or in none of the trials (No-Feedback). In Feedback trials, the selected target turned green for 700 ms when the cursor hit the target correctly and it turned red for 700 ms when hit incorrectly. In No-Feedback trials, the target never changed color when hit by the cursor. The pace of the task was rather fast, with each trial only lasting 1 s and trials within one block following each other without any break. A target turned light gray for the complete duration of the trial (1 s) or until it was hit by the cursor. For responses to be correct, a participant had to move from the middle of the screen to the target within a single second. Therefore, it was more difficult for a participant to establish whether the response as a whole was correct or incorrect without any feedback.

### Procedure

**Fig. 1:**
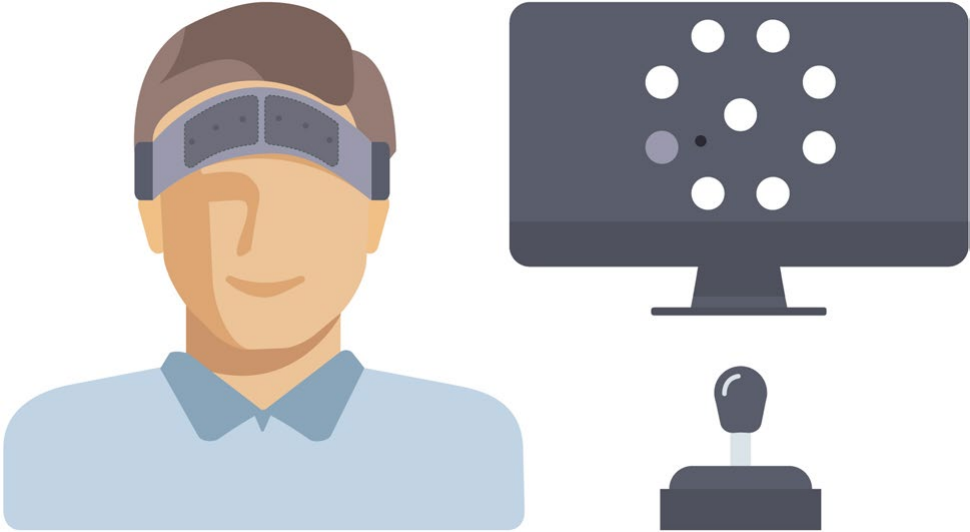
Schematic depiction of the placement of the two fNIRS sensor pads (left) above the eyebrows with the inside of both sensor pads aligned with the nasion and the learning task (right) with the joystick mounted on the table and the task presented on the monitor (cursor in black, the eight targets spaced at an equal distance from the middle with one selected target being darker)

After obtaining written and verbal informed consent, participants filled out a questionnaire on demographics. The fNIRS sensor pads were placed on the participants (Fig. 1, left), an eye tracker was calibrated, and data collection of the fNIRS device and eye tracker were started.^2^ First, participants sat calmly and still with their eyes fixated on a black fixation cross on a dark gray background for three minutes.

**Fig. 2:**
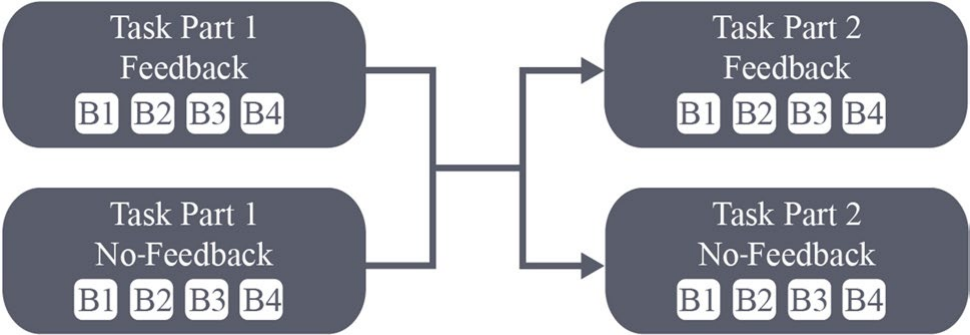
Schematic of the study design. Dark boxes depict the two parts of the task, in which ‘B’ in a lightbox stands for a block with 128 trials. In each part of the task participants either got direct feedback on their performance (Feedback) or not (No-Feedback)

Subsequently, the learning task (Fig. 1, right) was started for which participants were instructed to move the cursor using the joystick as fast and accurately as possible and to reverse sharply within the target circle back to the middle. Participants either performed No-Feedback trials or Feedback trials in both parts of the task (No Switch) or they performed No-Feedback trials in the first part of the task and Feedback trials in the second part of the task or the other way around (Switch). This resulted in four different groups of participants of which one group received feedback in both parts of the task, one did not receive feedback in both parts of the task, one only received feedback in the first (and not in the second) part of the task and one only received feedback in the second (and not in the first) part of the task. Participants were assigned to one of the groups using block randomization to take individual difference factors into account and to ensure every possible combination was used an equal number of times (Goodwin 2009). A schematic overview of the current study’s procedure is depicted in Fig. 2.

After completing the learning task, participants were asked to indicate the (most common) order in which the circles were selected as a target for each of the two parts of the task separately to test their declarative knowledge of the presented sequences, following Moisello et al. (2009) and Tinga et al. (2020a, b). Participants were instructed to guess if they did not recall the order. The experimental session, including setting up and removing the fNIRS sensors, took about 60–70 min per participant.

### Data processing and analyses

#### Processing of behavioral data

We computed multiple behavioral outcome measures to gain better insight into what aspects of the learning process are reflected in fNIRS outcome measures and followed Moisello et al. (2009) in computing a range of behavioral outcomes per trial: (1) whether the response on a trial was correct (i.e., when a movement was initiated from the middle and when the selected target was hit within 1 s); (2) time from the start of trial until the start of movement (onset time, OT); (3) time from the start of movement until the end of the movement (movement time, MT); (4) sum of the absolute OT and MT (response time, RT); (5) maximum speed of displacement between OT and end of the movement (peak velocity, PV); (6) linear distance from the endpoint of the movement and the center of the target (spatial error, SE), (7) the area in which the cursor moved divided by the squared movement length (normalized movement area, NMA).

#### Processing of fNIRS data

Collected fNIRS data were processed using the software program fnirSoft (Ayaz 2010). First, data were low-passed filtered with a finite impulse response filter with a cut-off frequency of 0.1 Hz to attenuate high-frequency noise and respiratory and cardiac noise (Izzetoglu et al. 2007). Next, a sliding-window motion artifact rejection (SMAR) algorithm was applied to eliminate motion artifacts and to reject problematic channels (Ayaz et al. 2010). Blood oxygenation and volume changes for each optode for each block were calculated using the Modified Beer–Lambert Law using a 3 s local baseline in the resting period before the start of each block. Neuronal activation induces an increase in metabolic demand and regional cerebral blood flow which causes an increase in oxygenated hemoglobin and a decrease in deoxygenated hemoglobin (Kamran et al. 2016). Changes in both oxygenated and deoxygenated hemoglobin are required for functional activation and therefore both indices are important to consider. Changes in oxygenation ([oxygenated hemoglobin] – [deoxygenated hemoglobin]; OXY) and total hemoglobin ([oxygenated hemoglobin] + [deoxygenated hemoglobin]; HbT) concentrations in micromolar (µM) were both calculated for further analyses, as both indices have been reported to be sensitive to training-related processes (Ayaz et al. 2012a, b). With increased cerebral blood flow HbT is thought to increase (Tachtsidis et al. 2009) and with increased oxygenation levels OXY is thought to increase (Gentili et al. 2013). As we expected PFC activity to decrease during learning, we expect both HbT and OXY to decrease over the current experiment’s learning task.

#### Statistical analyses

A declarative knowledge score was determined for each participant for each part of the task (i.e. Task Part 1 and Task Part 2) by computing the maximum overlap (in percentages) between the order of the sequence indicated by participants at the end of the experiment and the real sequence (of 16 targets).

Behavioral outcomes were averaged for each block. Averages for OT, MT, RT, PV, SE, and NMA were determined for correct trials only. Regarding the fNIRS data, average OXY and HbT changes over all four optodes were computed for each block.

To determine whether the block randomization was successful, we verified that the different groups of participants did not differ in age, gender and in baseline HBT and OXY by using logistic regression to test the effect of group on gender and using one-way ANOVAs to test the effect of group on age and on baseline HBT and OXY.

For all behavioral outcomes, we first tested the effect of the block on each outcome measure for each of the two parts separately. Subsequently, we tested the effect of task part on all behavioral outcome measures to assess learning over the whole experiment. The overall effect of the type of feedback (Feedback versus No-Feedback) was tested over all blocks. The effect of feedback on learning was tested through the interaction of task part with feedback. To assess the effect of (a switch in) feedback on learning we specifically examined the interaction with task part instead of task block as (switch in) type of feedback was manipulated for task parts.

Regarding the effects on the changes in OXY and HbT, we repeated the analyses on behavioral outcomes for these fNIRS outcomes to determine whether these outcomes change over the experiment during learning and what the effect of (a switch in) feedback is. Subsequently, it was tested whether effects on OXY and HbT were indeed specific to learning by examining the relationship overall blocks between these outcomes and behavioral outcomes.

All analyses on the effect of task part, block and feedback were performed with linear mixed-effects models analyses using *lme* in *R* (R Core Team 2017) with subject as random factor. The main effects of task part and block were tested with both whether feedback was provided and whether a switch in feedback occurred as random factors. Significant interactions were followed-up with post-hoc pairwise comparisons through applying the *R*-package *emmeans* with a Bonferroni correction to the mixed-effects models of the significant interactions.

## Results and discussion

Out of the 42 participants, 3 participants (7.14% of the data) were excluded, based on 2 criteria established in previous research. First, two participants achieved a declarative knowledge score above 40% (i.e., having a maximum overlap between the real and indicated order of 7 or more) in one of the two parts of the task. One participant achieved a declarative knowledge score of 43.75% in the first part of the task and the other achieved a declarative knowledge score of 68.75% in the second part of the task. These participants were excluded from further analyses, following the exclusion criteria also used by Curran and Keele (1993), Moisello et al. (2009), Tinga et al. (2020a, b) and Willingham et al. (1989). A declarative knowledge score of more than 40% is seen as significant and might influence the results as learning of the sequence would not be implicit for these participants (Moisello et al. 2009). Of the 40 remaining participants, the average declarative score was 14.88% (SD = 3.93%, range = 12.50–25.00%) and 15.31% (SD = 4.23%, range = 6.25–25.00%) for the first and second block respectively. A paired samples *t* test demonstrated no significant difference between the declarative knowledge scores in Part 1 and Part 2, *t* = 1.11, *p* = 0.269. Second, in 1 participant it could not be established that behavioral learning actually took place with performance deteriorating over time instead of improving. As an increase in behavioral performance over time would be evident in learning (Tinga et al. 2019) this participant was therefore removed from all further analyses.

The quality of the recorded fNIRS data was checked for the 39 remaining participants. The SMAR algorithm excluded data for one optode (out of four optodes) completely for four participants for all blocks. All other fNIRS data that remained were included for further analyses.

There were no significant differences between participants in the different groups in age (*p* = 0.537), gender (*p* = 0.322) and in baseline OXY (*p* = 0.234) and in baseline HBT (*p* = 0.803), demonstrating that the block randomization was successful.

### Behavioral effects

#### Learning effects

**Fig. 3:**
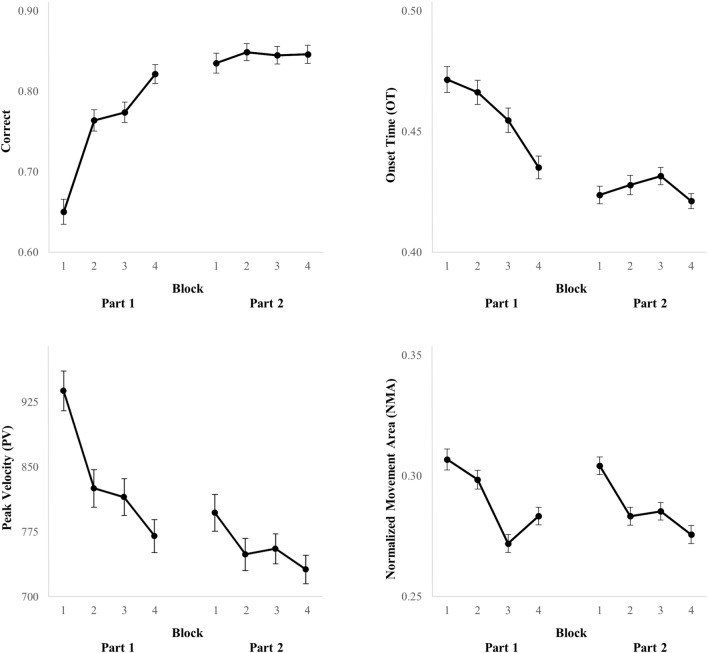
Overview of changes in correct responses (upper left, proportion correct), OT (upper right, seconds), PV (lower left, pixels per second), and NMA (lower right) per block. Error bars represent standard error of the mean

The details of all behavioral effects (*F*, *p*, *η*_*p*_^2^) over blocks in the first and second part of the task and from the first to the second part of the task are presented in Table 1 in columns 1–3. Specifics regarding statistically significant effects will be reported in this section. As an illustration of changes in behavioral performance, changes over blocks in correct responses, OT, PV and NMA are depicted in Fig. 3. As expected, performance improved over time during task learning. Correct responses increased by 17.13% within the first part of the task and by 9.12% from the first to the second part of the task, *F* = 56.51, *p* < 0.001, *η*_*p*_^2^ = 0.33 and *F* = 68.60, *p* < 0.001, *η*_*p*_^2^ = 0.20, respectively. OT decreased by 36.45 ms within the first part of the task and by 30.83 ms from the first to the second part of the task, *F* = 16.13, *p* < 0.001, *η*_*p*_^2^ = 0.12 and *F* = 43.75, *p* < 0.001, *η*_*p*_^2^ = 0.14 respectively. MT increased by 19.69 ms within the first part of the task and RT decreased by 28.12 ms from the first to the second part of the task, *F* = 21.80, *p* < 0.001, *η*_*p*_^2^ = 0.16 and *F* = 29.21, *p* < 0.001, *η*_*p*_^2^ = 0.10, respectively. PV decreased by 167.87 pixels per second within the first part of the task, *F* = 34.46, *p* < 0.001, *η*_*p*_^2^ = 0.23, by 65.88 pixels per second within the second part of the task, *F* = 10.96, *p* = 0.001, *η*_*p*_^2^ = 0.09, and by 78.90 pixels per second from the first to the second part of the task, *F* = 35.54, *p* < 0.001, *η*_*p*_^2^ = 0.12. SE decreased by 172.90 pixels within the first part of the task, *F* = 66.24, *p* < 0.001, *η*_*p*_^2^ = 0.36, by 20.52 pixels within the second part of the task, *F* = 4.53, *p* = 0.035, *η*_*p*_^2^ = 0.04 and by 77.32 pixels from the first to the second part of the task, *F* = 52.75, *p* < 0.001, *η*_*p*_^2^ = 0.16. NMA decreased with 2.35% within the first part of the task and with 2.85% within the second part of the task, *F* = 17.07, *p* < 0.001, *η*_*p*_^2^ = 0.13 and *F* = 20.18, *p* < 0.001, *η*_*p*_^2^ = 0.15, respectively. The results demonstrate that behavioral improvements mainly occurred within the blocks of the first part of the task and from the first to the second part of the task, with for example correct responses and OT improving clearly within Part 1, while outcome measures such as PV and NMA improved both during Part 1 and Part 2 as can be seen in Fig. 3. Although it could be expected that behavioral learning would take place across both task parts, it appears that most (i.e., 6 out of 7) behavioral outcome measures only improved within the first part of the task. Yet some behavioral learning also took place in the second part of the task as demonstrated by 3 out of 7 behavioral outcome measures also improving within Part 2. This finding does fit the results of Tinga et al. (2020b), a study whose design is most similar to the current study, in which the behavioral learning curve on a comparable SRT was also the steepest at the beginning of the task. Overall, over the course of the task behavioral performance became more correct with shorter movement onset times and total response times, while peak velocity decreased. Additionally, behavioral responses became more precise with both a decrease in spatial error and the total movement area.

**Table 1:** Behavioral effects (*F*, *p*, *η*_*p*_^2^) of feedback and task part and its interaction with feedback

Outcome measure	Block part 1		Block part 2		Task part		Feedback		Interaction feedback and task part	
	*F*	*η*_*p*_^2^	*F*	*η*_*p*_^2^	*F*	*η*_*p*_^2^	*F*	*η*_*p*_^2^	*F*	*η*_*p*_^2^
Number of correct responses (correct)	56.51***	0.33	0.41	0.00	68.60***	0.20	11.65***	0.04	4.93*	0.02
Onset time (OT)	16.13***	0.12	0.04	0.00	43.75***	0.14	6.87**	0.02	0.58	0.00
Movement time (MT)	21.80***	0.16	0.02	0.00	1.82	0.01	47.95***	0.14	4.18*	0.01
Response time (RT)	3.65	0.03	0.02	0.00	29.21***	0.10	24.63***	0.08	1.75	0.01
Peak velocity (PV)	34.46***	0.23	10.96**	0.09	35.54***	0.12	40.74***	0.13	0.73	0.00
Spatial error (SE)	66.24***	0.36	4.53*	0.04	52.75***	0.16	10.64**	0.03	3.17	0.01
Normalized movement area (NMA)	17.07***	0.13	20.18***	0.15	0.47	0.00	1.09	0.00	0.57	0.00

***p < 0.001, **p < 0.01, *p < 0.05

#### Effects of feedback

Details (*F*, *p, η*_*p*_^2^) for main effects of feedback and the interaction between task part and feedback effects on each behavioral outcome measure are presented in Table 1 in columns 4–6. In line with previous findings (Tinga et al. 2020a), feedback in general influenced overall behavioral performance. This effect was statistically significant for most of the behavioral outcome measures, these significant effects will be detailed in this section. Number of correct responses was 3.41% lower with Feedback than with No-Feedback, *F* = 11.65, *p* < 0.001, *η*_*p*_^2^ = 0.04. Additionally, OT was 6.40 ms higher, MT was 5.61 ms lower, and RT was 1.93 ms higher with Feedback, *F* = 6.87, *p* = 0.009, *η*_*p*_^2^ = 0.02, *F* = 47.95, *p* < 0.001, *η*_*p*_^2^ = 0.14 and *F* = 24.63, *p* < 0.001, *η*_*p*_^2^ = 0.08 respectively. PV was 113.89 pixels per second higher and SE was 44.29 pixels higher with Feedback, *F* = 40.74, *p* < 0.001, *η*_*p*_^2^ = 0.13 and *F* = 10.64, *p* = 0.001, *η*_*p*_^2^ = 0.03 respectively. These findings demonstrate that throughout the experiment feedback was associated with movements that started later but that were faster with a higher peak velocity. Yet, the spatial error was higher and responses were less correct with feedback, a finding that is in contrast to those demonstrating that feedback compared to no feedback enhances behavioral performance (Faulkner et al. 2011). The fact that feedback led to less correct responses and a higher spatial error in the current study can perhaps be explained by findings showing that trial-by-trial feedback might be distracting, especially when participants have a good sense of the task and their performance (Stanton and Young 2000), or by findings showing that feedback about the outcome of an action can reduce motivation (Kluger and Adler 1993).

In addition to the general effects of feedback, we also examined how learning was affected by feedback. Feedback interacted with task part for number of correct responses and MT, *F* = 4.93, *p* = 0.027, *η*_*p*_^2^ = 0.02 and *F* = 4.18, *p* = 0.042, *η*_*p*_^2^ = 0.01 respectively. Post-hoc pairwise comparisons demonstrated that the number of correct responses increased from Part 1 to Part 2 with Feedback or No-Feedback in both parts and with Feedback in Part 1 and No-Feedback in Part 2, all *t* ≥ 2.78 and all *p* < 0.035. Yet, the number of correct responses did not increase when No-Feedback was presented in Part 1 and Feedback in Part 2, *t* = 2.02, *p* = 0.266. Additionally, participants had more correct responses in Part 1 with No-Feedback compared to Feedback, *t* = 4.05, *p* < 0.001, yet no differences in correct responses between No-Feedback and Feedback were found in Part 2, *t* = 0.82, *p* = 1.000. These findings demonstrate that responses became more correct over blocks unless there was a switch from No-Feedback in Part 1 to Feedback in Part 2, which might have been caused by responses being more correct in Part 1 when no feedback was provided. Regarding MT, post-hoc pairwise comparisons showed a significant difference for all 4 possible comparisons between Feedback and No-Feedback, all *t* ≥ 3.21 and all *p* ≤ 0.009, but with no difference between Part 1 with Feedback and Part 2 with Feedback and no difference between Part 1 with No-Feedback and Part 2 with No-Feedback, all *t* < 2.45 and all *p* ≥ 0.09. These findings reflect that MT was higher with No-Feedback in general, but that there was no significant decrease in MT from Part 1 to Part 2 when there was no switch in the type of feedback.

### fNIRS effects

#### Learning effects

**Fig. 4:**
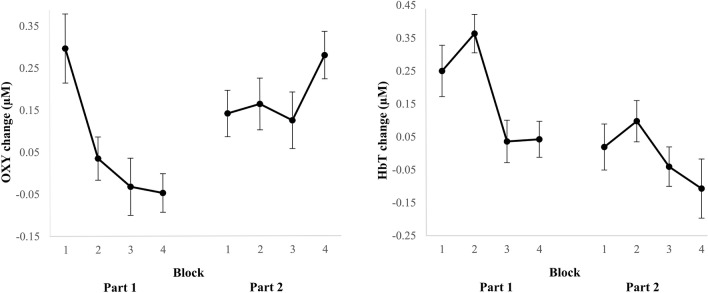
Overview of changes in OXY (left) and HbT (right) per block. Error bars represent standard error of the mean

The average OXY and HbT changes per block are depicted in Fig. 4. The details of all effects (*F, p, η*_*p*_^2^) over blocks on OXY and HbT in the first and second part of the task and from the first to the second part of the task are presented in Table 2 in columns 1–3. Specifics regarding statistically significant effects will be reported in this section. OXY decreased with 0.34 µM within the first part of the task, *F* = 4.56, *p* = 0.035, *η*_*p*_^*2*^ = 0.04. HbT decreased with 0.21 µM within the first part of the task and with 0.18 µM from the first to the second part of the task, *F* = 4.51, *p* = 0.036, *η*_*p*_^2^ = 0.04 and *F* = 5.22, *p* = 0.023, *η*_*p*_^2^ = 0.02 respectively. Even though OXY seems to change within the second part of the task (see Fig. 4), effects over blocks within this second part and from the first to the second part were not significant. The significant changes in fNIRS outcomes suggest that less cognitive effort needed to be exerted overtime during learning. As HbT decreased both within the first part of the task and over the two task parts, HbT might be more sensitive to learning than OXY.

**Table 2:** fNIRS effects (*F*, *p*, *η*_*p*_^2^) of block in part 1 and 2, task part, feedback and the interaction of task part with feedback

Outcome measure	Block part 1		Block part 2		Task part		Feedback		Interaction feedback and task part		
	*F*	*η*_*p*_^2^	*F*	*η*_*p*_^2^	*F*	*η*_*p*_^2^	*F*	*η*_*p*_^2^	*F*		*η*_*p*_^2^
Oxygenation (OXY)	4.56*	0.04	0.73	0.01	2.29	0.01	0.01	0.00	0.86		0.00
Total hemoglobin (HbT)	4.51*	0.04	0.97	0.01	5.22*	0.02	1.19	0.00	0.00		0.00

*p < 0.05

#### Effects of feedback

Details (*F*, *p, η*_*p*_^2^) for main effects of feedback and the interaction between task part and feedback effects on both OXY and HbT are presented in Table 2 in columns 4–6. Both fNIRS outcomes were not sensitive to feedback. This finding suggests that fNIRS is unaffected by task feedback.

#### Relationship with behavioral outcome measures

Details (*η*_*p*_^2^) for all effects on the relationship between the fNIRS outcome measures and behavioral performance overall blocks are presented in Table 3. Specifics regarding statistically significant effects will be reported in this section. A decrease in HbT was related to an increase in correct responses and a decrease in SE, *F* = 11.50, *p* < 0.001, *η*_*p*_^2^ = 0.04 and *F* = 12.34, *p* < 0.001, *η*_*p*_^2^ = 0.04 respectively. Although HbT was related to behavioral performance, no such relationship was found for OXY. These results suggest that changes in HbT (but not OXY) are coinciding with changes in behavioral performance. Considering the finding that HbT was also more sensitive to changes over time than OXY, HbT seems to be a more suitable outcome measure for measuring learning effects in the current task.

**Table 3:** fNIRS explanatory power (*η*_*p*_^2^) in behavioral outcome measures

Outcome measure	*η*_*p*_^2^ Correct	*η*_*p*_^2^ OT	*η*_*p*_^2^ MT	*η*_*p*_^2^ RT	*η*_*p*_^2^ PV	*η*_*p*_^2^ SE	*η*_*p*_^2^ NMA
Oxygenation (OXY)	0.00	0.00	0.00	0.00	0.00	0.00	0.01
Total hemoglobin (HbT)	0.04***	0.00	0.00	0.00	0.01	0.04***	0.01

***p < .001

## General discussion

fNIRS is a promising technique for assessing PFC activity in learning (Leff et al. 2011). As changes in brain activity during learning are thought to be affected by (a change in) whether feedback is provided or not (Tinga et al. 2019), brain activity as assessed through fNIRS might potentially be sensitive to feedback. Yet, only a few studies have examined fNIRS outcomes in learning and studies additionally examining the effects of feedback are non-existent. To address this gap in the literature, the goal of the current study was to examine PFC activity measured through fNIRS during visuomotor learning and to examine how results are affected by feedback and a change in feedback.

Activity in the PFC as measured through fNIRS significantly decreased within the first and second part of the task and from the first to the second part. This finding is consistent with results of previous studies on fNIRS specifically (Ayaz et al. 2012a, b; Leff et al. 2011; Sagari et al. 2015; Tinga et al. 2019) and previous studies on EEG (Tinga et al. 2019, 2020a) and suggests that less cognitive effort needed to be exerted over the course of learning. Additionally, changes in PFC activity were related to behavioral performance, suggesting that these changes coincide with behavioral learning, which fits with previous studies demonstrating a relationship between behavioral performance and fNIRS specifically during visuomotor learning (Ayaz et al. 2012a) and brain activity as measured through EEG (Hamame et al. 2011; Moisello et al. 2013; Nikolaev et al. 2016; Tan et al., 2016; Tinga et al. 2019, 2020b).

In the current study, learning effects were more pronounced on HbT than on OXY, suggesting that HbT might be more sensitive to learning than OXY. A previous study by Ayaz et al. (2012a) employing fNIRS in the PFC to study visuomotor learning during a flight simulator task only reported changes in HbT, while another previous study by Ayaz et al. (2012b) only reported changes in PFC in OXY during learning of a similar flight simulator task. While Harrison et al. (2014) explicitly reported computing both OXY and HbT changes in PFC during air traffic monitoring learning, the authors only presented results on OXY, with changes occurring over the course of learning. Thus, although both OXY and HbT are features computed based on oxygenated and deoxygenated hemoglobin, it is still unclear which of the two features is most suitable for gaining insight into learning. HbT is mostly related to cerebral blood flow, while OXY is mostly related to oxygenation levels (Gentili et al. 2013; Tachtsidis et al. 2009), with changes in brain activity inducing changes in both OXY and HbT. We recommend future studies to include both features to contribute to establishing which one is the most suitable under what circumstances.

Changes in both OXY and HbT during learning were unaffected by feedback. This result is consistent with findings on EEG in our previous experiment (Tinga et al. 2020a) in which changes in EEG activity over the course of visuomotor learning were also unaffected by feedback. Yet, these findings are in contrast to the finding demonstrated through meta-analysis that neurophysiological changes, including changes in brain activity, during learning are affected by feedback (Tinga et al. 2019) and to findings from experimental work demonstrating EEG is affected by different types of feedback (Arbel et al. 2014; Fairclough and Roberts 2011; Opitz et al. 2011; Venables and Fairclough 2009). Although the findings of the current study suggest that PFC activity measured through fNIRS during visuomotor learning is unaffected by feedback, it might be the case that different types of feedback manipulation would have different effects. For example, the current study presented participants both with positive and negative feedback for correct and incorrect responses respectively. Feedback reflecting actual performance obviously has more ecological validity and it has been demonstrated that it enhances the learning of new (motor) skills compared to ‘false’ feedback not reflecting actual task performance (Hirst et al. 2013; Mackworth 1964; Palmer et al. 2015). Yet, as previous research established different effects on EEG of positive versus negative feedback while generally presenting participants with false feedback (Arbel et al. 2014; Fairclough and Roberts 2011; Venables and Fairclough 2009), effects of feedback on fNIRS could be different when presenting either only false positive or negative feedback not reflective of actual task performance.

Another aspect possibly affecting the fNIRS results in the current study may be the fact that behavioral performance primarily improved in the first part of the learning task, while improvements were less evident in the second part of the task. Effects of learning on fNIRS outcome measures might be more pronounced with a steep learning curve throughout the complete learning task. Additionally, behavioral performance was less correct with an increased spatial error with feedback compared to no feedback. Feedback has the potential to improve behavioral performance (Faulkner et al. 2011) and the effects of feedback leading to improvements could have different effects on fNIRS outcome measures. Therefore, future studies may examine effects on fNIRS outcome measures with a different study design attaining different effects on behavioral performance.

Moreover, while the current study only examined PFC activity, changes during visuomotor learning have also been reported with fNIRS in other brain areas, such as the supplementary motor area and the pre-supplementary motor area (Hatakenaka et al. 2007; Sagari et al. 2015) and the sensory-motor cortex (Hatakenaka et al. 2007; Hiyamizu et al. 2014; Ikegami and Taga 2008; Sagari et al. 2015). Effects of visuomotor learning on brain activity are not only reflected in reduced activity suggesting a decrease in the cognitive effort; enhanced brain activity during learning has also been demonstrated. For example, activity in the supplementary motor area has been demonstrated to increase during visuomotor learning (Hatakenaka et al. 2007; Hiyamizu et al. 2014), which is thought to be related to skilled motor execution in particular (Hatakenake et al. 2007). Therefore, an interesting endeavor for future work is to examine other cortical brain areas as well and to examine whether learning as assessed through fNIRS in other cortical areas is affected by task feedback reflecting actual performance.

The current study is the first to explore PFC activity measured through fNIRS during visuomotor learning and the effects of task feedback. All in all, the findings demonstrate that fNIRS in the PFC is valuable for assessing visuomotor learning and that this measure is robust to task feedback. The current study highlights the potential of fNIRS in assessing learning even under different task feedback conditions and when changes in task feedback occur.

## Data Availability

The datasets generated during and/or analyzed during the current study are not publicly available as the ethical committee did not grant permission to do so due to the fact that sensitive physiological data was collected in the current experiment.

## Footnotes

1 Concluded based on a literature search conducted in the Web of Science database on November 5th 2019 using a combination of the search terms ‘learning’, ‘feedback’, ‘*NIR*’ and “near infrared spectroscopy” in addition to searching through all the cited literature in the current manuscript.

2 For the purpose of the current study, data collected by the eye tracker was not included.
